# Supplementary material for: From sequence to enzyme mechanism using multi-label machine learning
Source: BMC Bioinformatics. 2014 May 19;15:150. doi: 10.1186/1471-2105-15-150 (PMC4229970; doi:10.1186/1471-2105-15-150)
Supplement: Additional file 2 — Java code of ml2db. Additional file ml2db_code.tar.gz contains the Java source code to run the multi-label machine learning experiments and save the results to database. The code’s Javadoc is included. [file 1471-2105-15-150-S2.zip › additional file 2/ml2db/ml2db/doc/index-files/index-14.html]

N-Index


JavaScript is disabled on your browser.


- Overview
- Package
- Class
- Use
- Tree
- Deprecated
- Index
- Help

- Prev Letter
- Next Letter

- Frames
- No Frames

- All Classes

A B C D E F G H I J K L M N O P Q R S T U V W X 


## N

N1 - Static variable in class main.ml2db.mulan.learn.MulanLearners


NAME - Static variable in class main.ml2db.al.utility.confidence.ConfidenceMetric


NAME - Static variable in class main.ml2db.al.utility.globaldistance.GlobalDistanceMetric


NAME - Static variable in class main.ml2db.al.utility.hybrid.GlobalDistanceWithConfidenceMetric


NearestNeighboursByFreqUtility - Class in main.ml2db.al.utility.pickers.byfrequency
:   Class

NearestNeighboursByFreqUtility() - Constructor for class main.ml2db.al.utility.pickers.byfrequency.NearestNeighboursByFreqUtility


NearestNeighboursByFreqUtilityTest - Class in test.ml2db.al.utility.neighbourhood
:   Class

NearestNeighboursByFreqUtilityTest() - Constructor for class test.ml2db.al.utility.neighbourhood.NearestNeighboursByFreqUtilityTest


Neighbourhood - Class in main.ml2db.al.utility.neighbours
:   Contains information about: an instance, how much it is needed in this data
    set (the instances it is the nearest neighbour of), and how much it is
    labelled (whether it or its nearest or further neighbours are already
    labelled or partially known).

Neighbourhood(String, Instance) - Constructor for class main.ml2db.al.utility.neighbours.Neighbourhood


NEIGHBOURHOOD\_BASE\_NAME - Static variable in class main.ml2db.al.utility.pickers.byfrequency.NearestNeighboursByFreqUtility


NeighbourhoodExperimentDetails - Class in main.ml2db.al.utility.neighbours
:   Class

NeighbourhoodExperimentDetails() - Constructor for class main.ml2db.al.utility.neighbours.NeighbourhoodExperimentDetails


NeighbourhoodExperimentDetailsSerialised - Class in main.ml2db.al.utility.neighbours
:   Class that uses an already calculated neighbourhood network to run an active
    learning experiment.

NeighbourhoodExperimentDetailsSerialised() - Constructor for class main.ml2db.al.utility.neighbours.NeighbourhoodExperimentDetailsSerialised


NeighbourhoodExperimentDetailsTest - Class in test.ml2db.al.utility.neighbourhood
:   Class

NeighbourhoodExperimentDetailsTest() - Constructor for class test.ml2db.al.utility.neighbourhood.NeighbourhoodExperimentDetailsTest


NeighbourhoodNetwork - Class in main.ml2db.al.utility.neighbours
:   A collection of instances "needed" to label other instances.

NeighbourhoodNetwork(IdMultiLabelInstances) - Constructor for class main.ml2db.al.utility.neighbours.NeighbourhoodNetwork
:   a list of needed instances

NeighbourhoodNetworkTest - Class in test.ml2db.al.utility.neighbourhood
:   Class

NeighbourhoodNetworkTest() - Constructor for class test.ml2db.al.utility.neighbourhood.NeighbourhoodNetworkTest


NeighboursPrinter - Class in main.ml2db.mulan.mulanarff
:   Class to store the neighbours of an instance and print them in a
    human/graphviz readable format.

NeighboursPrinter(IdMultiLabelInstances, Set<String>) - Constructor for class main.ml2db.mulan.mulanarff.NeighboursPrinter


NeighboursPrinterTest - Class in main.ml2db.mulan.mulanarff
:   Class

NeighboursPrinterTest() - Constructor for class main.ml2db.mulan.mulanarff.NeighboursPrinterTest


neighboursToString() - Method in class main.ml2db.mulan.mulanarff.NeighboursPrinter


NOMINAL - Static variable in class main.ml2db.weka.AttributeFactory


NUMBER\_OF\_ATTRIBUTES - Static variable in class main.ml2db.weka.ArffProps
:   the total number of possible attributes for the dataset

NUMBER\_OF\_CLASS\_VALUES - Static variable in class main.ml2db.weka.ArffProps
:   the total number of possible class values for the dataset

NUMBER\_OF\_CORE\_PROPERTIES - Static variable in class main.ml2db.weka.ArffProps
:   the number of properties that should be available on the properties file
    (check)

NUMBER\_OF\_INSTANCES - Static variable in class main.ml2db.weka.ArffProps
:   the number of instances (rows) in the arff file

NUMBER\_OF\_INSTANCES\_IN\_TEST\_ARFF - Static variable in class test.ml2db.dataharness.DataOne


NUMBER\_OF\_INSTANCES\_IN\_TEST\_ARFF - Static variable in class test.ml2db.dataharness.DataTwo


NUMBER\_OF\_NEIGHBOURS\_1 - Static variable in class main.ml2db.al.utility.neighbours.NeighbourhoodNetwork


NUMBER\_OF\_NEIGHBOURS\_1 - Static variable in class main.ml2db.mulan.mulanarff.NeighboursPrinter


NUMBER\_OF\_STATISTICS\_PROPERTIES - Static variable in class main.ml2db.weka.ArffProps
:   the number of properties + number of data statistics (check: should be 9
    + 3)

numberOfUnlabelledNeedyInstances(String) - Method in class main.ml2db.al.utility.neighbours.NeighbourhoodNetwork


NUMERIC - Static variable in class main.ml2db.weka.AttributeFactory

A B C D E F G H I J K L M N O P Q R S T U V W X

- Overview
- Package
- Class
- Use
- Tree
- Deprecated
- Index
- Help

- Prev Letter
- Next Letter

- Frames
- No Frames

- All Classes
